# Mechanosensitive traction force generation is regulated by the neutrophil activation state

**DOI:** 10.1038/s41598-023-37997-y

**Published:** 2023-07-09

**Authors:** Hadley Witt, Zicheng Yan, David Henann, Christian Franck, Jonathan Reichner

**Affiliations:** 1 Graduate Program in Pathobiology, Brown University, Providence, RI 02912 USA; 2 Division of Surgical Research, Department of Surgery, Rhode Island Hospital, Providence, RI 02903 USA; 3 School of Engineering, Brown University, Providence, RI 02912 USA; 4 Department of Mechanical Engineering, University of Wisconsin-Madison, Madison, WI 53706 USA

**Keywords:** Experimental models of disease, Deformation dynamics, Biophysical methods

## Abstract

The generation of traction forces by neutrophils regulates many crucial effector functions responsible for host defense, such as attachment, spreading, migration, phagocytosis, and NETosis. The activation state of the cell is a strong determinant of the functional efficacy of the neutrophil; however, the effect of activation on traction force production has not yet been determined experimentally. Previously, the mapping of cellular-generated forces produced by human neutrophils via a Traction Force Microscopy (TFM) method has required a three-dimensional imaging modality to capture out-of-plane forces, such as confocal or multiphoton techniques. A method newly developed in our laboratories can capture out-of-plane forces using only a two-dimensional imaging modality. This novel technique—combined with a topology-based single particle tracking algorithm and finite element method calculations—can construct high spatial frequency three-dimensional traction fields, allowing for traction forces in-plane and out-of-plane to the substrate to now be differentially visualized and quantified with a standard epifluorescence microscope. Here we apply this technology to determine the effect of neutrophil activation on force generation. Sepsis is a systemic inflammatory response that causes dysregulated neutrophil activation in vivo. We found that neutrophils from septic patients produced greater total forces than neutrophils from healthy donors and that the majority of this dysregulation occurred in-plane to the substrate. Ex vivo activation of neutrophils from healthy donors showed differential consequences depending on activation stimuli with mechanosensitive force decreases observed in some cases. These findings demonstrate the feasibility of epifluorescence-based microscopy in mapping traction forces to ask biologically significant questions regarding neutrophil function.

## Introduction

Neutrophils are the most abundant circulating white blood cell in the human body and the first responders to tissue injury and infection. During inflammation, neutrophils are recruited to the site of tissue affliction to mediate the clearance of pathogens, begin the wound healing process, and return the tissue to homeostasis^1–4^. Neutrophils circulate in the healthy host in a naive/resting state and undergo cellular activation upon exposure to damaged tissue and/or microbial components^3–5^. Neutrophil activation regulates essential host defense functions including degranulation, NETosis, phagocytosis, spreading, and migration. Therefore, the activated neutrophil functions at a heightened capacity in host defense^6,7^.

When systemic and non-specific neutrophil recruitment and activation occur, such as during sepsis, the result can be damage to the tissue microvascular and parenchymal cells, leading to possible organ failure and death^6,8–12^. Although many studies have examined the role of neutrophil activation on effector functions, a focused examination of the role of activation on neutrophil-generated forces has not yet been reported. This represents a gap in our understanding of neutrophil regulation by priming and/or activation, because effector functions essential for host defense also depend on force production.

Bodily tissue can be described as a highly elastic substrate against which neutrophils produce traction forces to migrate to the precise source of infection and execute antimicrobial effector functions^13–15^. This tissue elasticity is in contrast to the rigid surfaces of tissue culture plastic on which most laboratory investigations are conducted. These physical or “mechanical” properties of tissue are now understood to exert regulatory effects on cells, a phenomenon characterized in the field of mechanobiology^16–23^. Human neutrophils are highly sensitive to the mechanical property of substrate stiffness, exhibiting differential spreading, motility, and traction force generation^14,24–27^. The differential regulation of neutrophil traction and migration as a function of substrate stiffness is significant as it suggests that neutrophils entering an afflicted tissue as soft and elastic as the brain^28–30^ might generate forces with different kinetics and modalities than neutrophils entering less pliable tissues such as liver or muscle^31–34^. However, the effect of neutrophil activation on mechanosensitivity is not well defined.

Neutrophils attach and spread to their underlying substrate through the generation of traction forces, which is a complex cellular process that can be visualized and quantified via Traction Force Microscopy (TFM)^5,14,24,35–37^. As neutrophils generate forces against tissue and extracellular matrix, they physically deform their underlying substrate. TFM is performed by embedding fluorescent beads into the hydrogel substrate and comparing bead locations before and after cells are present or stimulated^14,24,35,36^. This comparison is used to calculate the displacement caused by the cell and-taken together with the substrate stiffness-indicates the traction forces produced by that cell. In order to visualize three-dimensional (3D) deformation fields, beads are often fully embedded throughout the substrate material, and therefore require a 3D imaging modality-such as such as confocal or multiphoton techniques-to visualize out-of-plane displacements and to account for the spatial resolution requirements^35^. In addition to a 3D imaging modality, these techniques also involve high computational costs, which greatly limits their usability and accessibility.

Recently, our laboratory developed a novel TFM technique for the visualization of 3D displacements with low computation costs and using only epifluorescence microscopy (Supp. Fig. 1)^38^. By utilizing a rapid, topology-based particle tracking (T-PT) method that can track high density fiducial markers along with highly complex and large deformations, beads can now be in a single planar conformation, as opposed to full 3D embedment (Supp. Fig. 2)^39^. This single layer of beads therefore allows the capture of 3D deformation fields from an epifluorescence microscope as opposed to the need for more complex imaging techniques by confocal microscopy (Supp. Fig. 3). Finite element method (FEM) calculations were then used to convert displacement fields captured using epifluorescence into 3D traction fields. In addition to quantifying total 3D displacements and traction forces, this technology also permits the decoupling of cell-generated tractions in-plane to the substrate from tractions out-of-plane to the substrate. This method is supported with the commercial software package Abaqus. Overall, traction force microscopy of a single-layer of high-density beads using the novel tracking method T-PT combined with finite element method calculations can visualize 3D tractions on an epifluorescence microscope with greatly reduced computational costs. As such, this method marks the next technological step for high-accessibility traction measures^38^. We show here for the first time the biological application of this new technology to determine how the activation state of neutrophils regulates the generation of traction forces.

## Results

### Dysregulation of forces and tractions in neutrophils from septic donors

**Figure 1:**
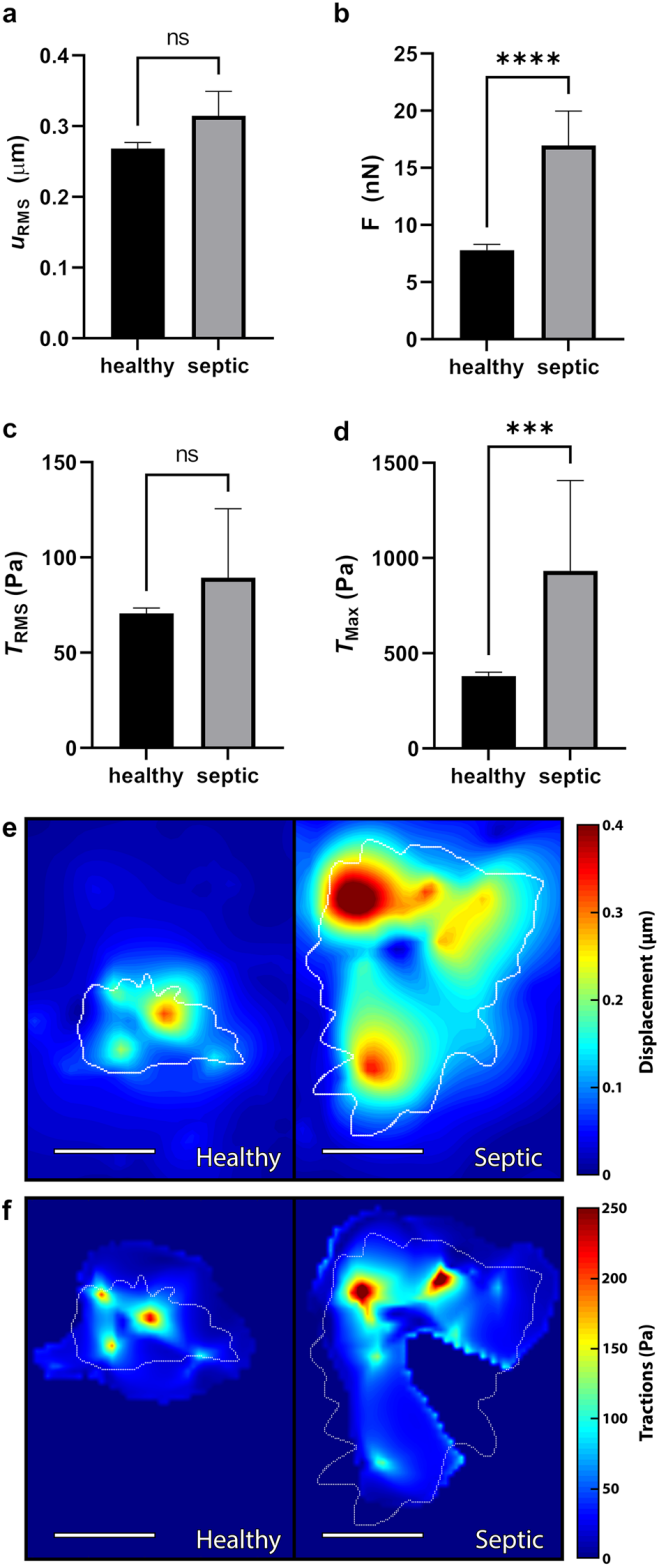
Neutrophils from septic patients produce greater forces and traction maxima than neutrophils from healthy donors. Traction force microscopy of unstimulated neutrophils from either a healthy donor (n = 293) or septic donor (n = 24) on 1.5 kPa polyacrylamide gel substrates, quantifying mean and standard error mean (SEM) of (**a**) root-mean-square displacement ($u_{RMS}$), (**b**) total force (${\textbf {F}}$), (**c**) root-mean-square traction ($T_{RMS}$), and (**d**) traction maxima ($T_{Max}$). Cells were maintained at $37\;^{\circ }\text {C}$ throughout the duration of the experiment. Displacements were computed in Matlab by T-PT, and displacement heatmaps $|{\textbf {u}}|$ (**e**) and traction heatmaps $|{\textbf {t}}|$ (**f**) were generated for two representative cells at 30 min; forces and tractions were computed using the finite element method in Abaqus. Scale bars are 10 μm, and the white contours represents the boundary of the cell edge. ***p < 0.001, ****p < 0.0001 (unpaired Student’s t-test).

To gain insight into neutrophil dysregulation during sepsis, we compared how neutrophils from healthy donors and neutrophils from septic donors differentially displace their underlying substrate. Neutrophils were isolated from whole blood and were then seeded on soft hydrogels with small fluorescent fiducial markers embedded just below the surface. As the neutrophils attached and migrated, deforming the hydrogel surface, these markers were displaced. Volumetric imaging of these regions before and after cell attachment were then quantified to calculate root-mean-square displacement ($u_{RMS}$) by the cell; this showed no statistically significant difference of average substrate displacement between neutrophils from healthy and septic donors (Fig. 1a). We next looked at the total forces (${\textbf {F}}$)^24^ exerted by the cells to create these substrate displacements and found that neutrophils from septic donors generate greater total forces than neutrophils from healthy donors (Fig. 1b). Next, to remove the variable of spread area, we looked at root-mean-square tractions ($T_{RMS}$)—which is an average magnitude of the traction field across the surface of the cell-substrate interface—and found that neutrophils from healthy and septic donors were comparable, indicating that an increase in spread area, not an increase in average tractions, accounted for the differential healthy and septic force phenotype (Fig. 1c).

Finally, we looked at the traction maxima ($T_{Max}$) of these neutrophils—which is the highest traction magnitude at a single point due to a given cell—and found that cells from septic donors induced much higher $T_{Max}$ than cells from healthy donors (Fig. 1d). Taken together, these findings suggest that while the average displacements and traction forces from neutrophils of septic donors are comparable to healthy donors, these cells are much more spread—therefore in much greater contact with the substrate—and thus produce greater total forces overall. Further, the septic dysregulation in $T_{Max}$ indicates that while the traction forces of neutrophils from healthy donors are more evenly distributed across the cell-substrate interface, neutrophil traction forces during sepsis are much more punctate and irregular. This can be further visualized by contour plots of the displacement (Fig. 1e) and traction magnitude fields (Fig. 1f).

**Figure 2:**
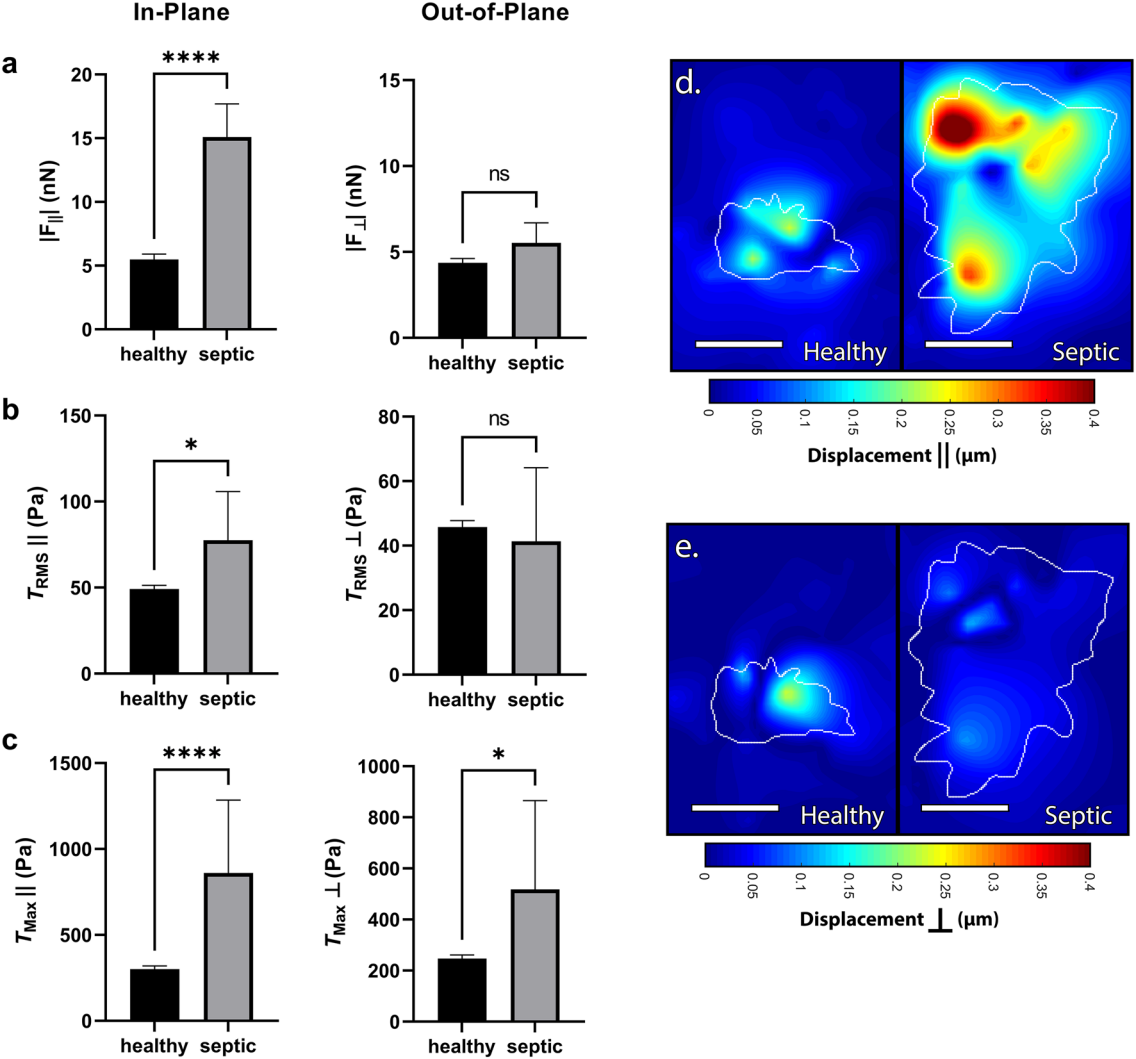
Dysregulation of force production from septic neutrophils occurs primarily in-plane. Traction force microscopy of neutrophils from a healthy donor (n = 293) or a septic donor (n = 24) on 1.5 kPa polyacrylamide gels wherein the in-plane traction component ($\parallel$) along the surface of the substrate is separated from the out-of-plane traction component ($\perp$) going into/out-of the surface of the substrate, computing mean (and SEM) of (**a**) in-plane total forces (${\textbf {F}}_{\parallel }$) and out-of-plane total forces (${\textbf {F}}_{\perp }$), (**b**) in-plane RMS tractions ($T_{RMS}\parallel$) & out-of-plane RMS tractions ($T_{RMS}\perp$), and (**c**) in-plane traction maxima ($T_{Max}\parallel$) and out-of-plane traction maxima ($T_{Max}\perp$). Displacements were computed in Matlab by T-PT, and in-plane (**d**) and out-of-plane (**e**) displacement heatmaps were generated for two representative cells at 30 min; tractions were computed using the finite element method in Abaqus. Scale bars are 10 μm, and the white contours represents the boundary of the cell edge. *p < 0.05, ****p < 0.0001 (unpaired Student’s t-test).

Neutrophils generate traction forces in two modes: (1) in-plane to the substrate ($\parallel$, along the surface) and (2) out-of-plane to the substrate ($\perp$, into/out-of the surface), which are differentially regulated^24^. Therefore, we next sought to determine the planar distribution in force generation during septic dysregulation. We found that nearly all of the extraneous forces and tractions from septic patients occur in-plane to the surface, indicating a highly directional dysregulation during septic motility and contractility (Fig. 2a–e). While out-of-plane total forces (${\textbf {F}}_{\perp }$) were comparable between septic and healthy donors, septic donors generated greater in-plane total forces (${\textbf {F}}_{\parallel }$) as compared to healthy (Fig. 2a). Similarly, in-plane RMS tractions ($T_{RMS}\parallel$) were greater in septic donors compared to healthy donors, while out-of-plane RMS tractions ($T_{RMS}\perp$) were comparable between both groups (Fig. 2b). However, the in-plane and out-of-plane traction maxima ($T_{Max}\parallel$ and $T_{Max}\perp$) were both increased for septic donors (Fig. 2c). These findings indicate a directional component to neutrophil traction dysregulation with most of the extraneous force occurring in-plane to the substrate, which can be further visualized in the displacement heatmaps (Fig. 2d,e).

Taken together, these findings suggest that while root-mean-square displacements and tractions generated by neutrophils from septic donors are comparable to healthy donors, these cells are much more spread and thus produce greater total forces overall. Further, the septic dysregulation in $T_{Max}$ indicates that while the traction forces of neutrophils from healthy donors are more evenly distributed across the cell-substrate interface, neutrophil traction forces during sepsis are much more punctate and irregular. Finally, while neutrophils from septic donors have a larger spread area and more irregular force distribution, most of this dysregulation occurs in-plane to the substrate.

### Differential traction forces mediated by ex vivo activation

**Figure 3:**
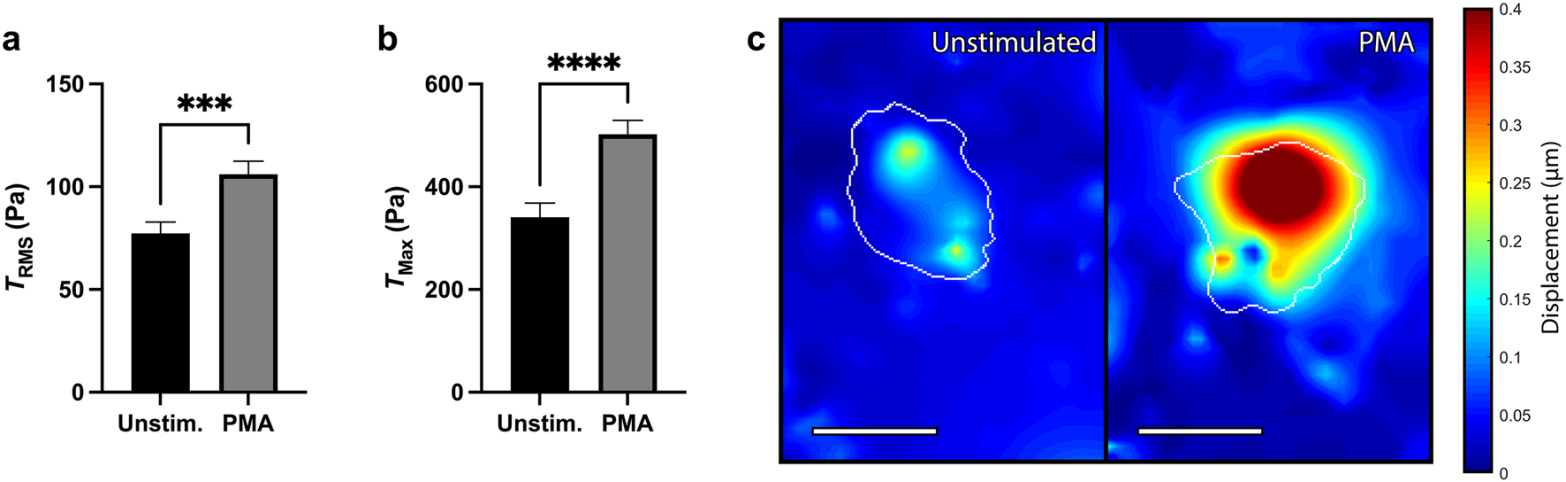
RMS tractions and traction maxima of neutrophils from healthy donors after stimulation by PMA. Mean (and SEM) of (**a**) root-mean-square tractions ($T_{RMS}$) and (**b**) traction maxima ($T_{Max}$) of neutrophils from healthy donors seeded on 1.5 kPa polyacrylamide gels (n = 70). Cells were allowed to attach and settle and then imaged before stimulation (“Unstim.”). Cells were then treated with 20nM Phorbol Myristate Acetate (“PMA”) and imaged 30 min post-addition. Cells were maintained at 37 °C throughout the duration of the experiment. Displacements were computed in Matlab by T-PT, and displacement heatmaps were generated for a representative cell at 30 min (**c**); tractions were computed using the finite element method in Abaqus. Scale bars are 10 μm, and the white contours represents the boundary of the cell edge. ***p < 0.001, ****p < 0.0001 (paired Student’s t-test).

We next examined the role of ex vivo activation on neutrophil-induced traction force generation. Neutrophils were isolated from healthy donors and seeded on soft hydrogels as described above. Cells were imaged prior to activation to get their unstimulated state and then activated with Phorbol Myristate Acetate (PMA) for 30 min; PMA was chosen due to its ability to strongly stimulate neutrophils through a receptor-independent mechanism. Activation by PMA greatly increased neutrophil-generated $T_{RMS}$ (Fig. 3a). There was also an increase in $T_{Max}$ generation (Fig. 3b), as well as displacement of the underlying substrate (Fig. 3c).

**Figure 4:**
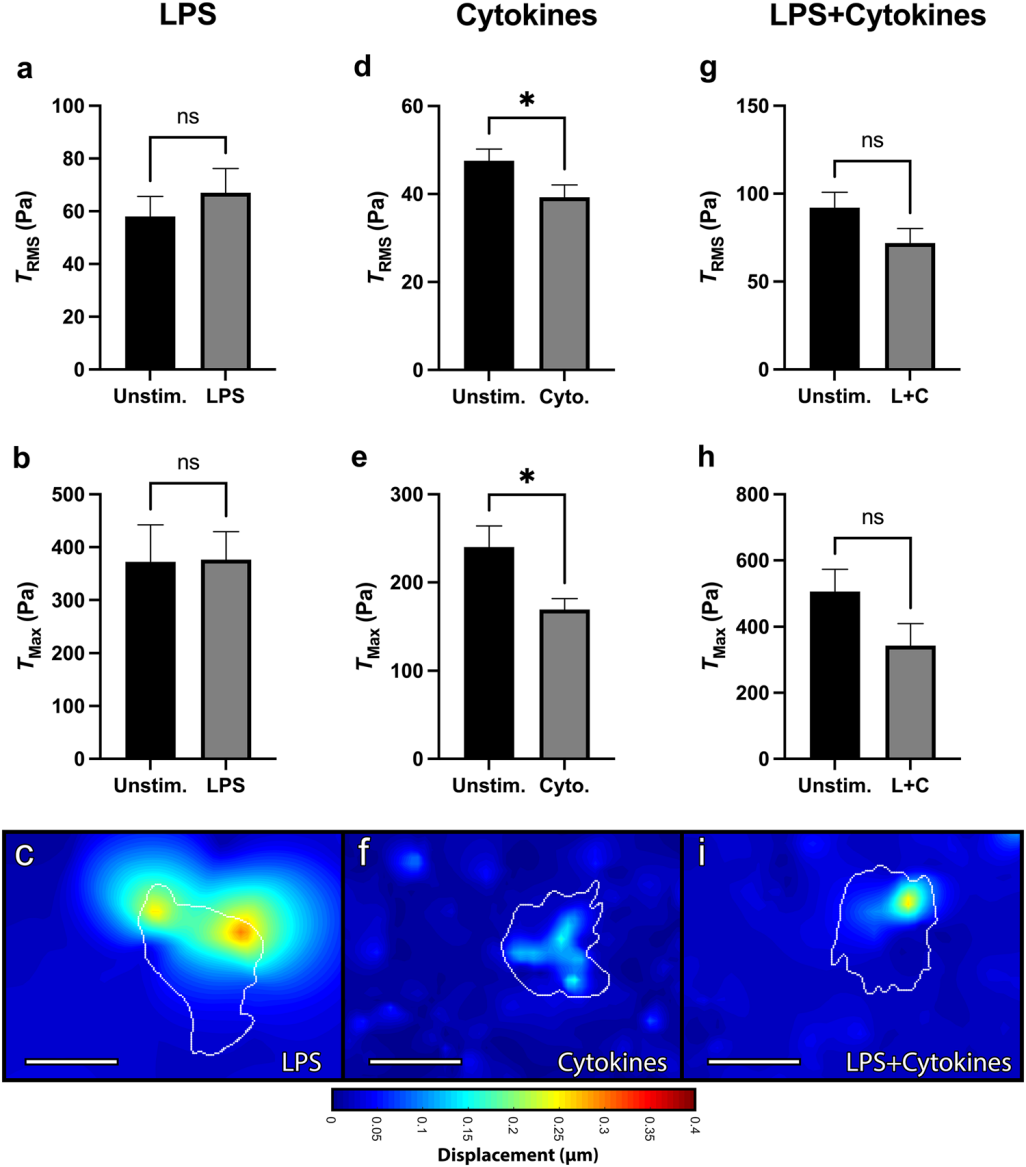
RMS tractions and traction maxima of neutrophils from healthy donors after stimulation by LPS, IFN-$\gamma$, and GM-CSF. Mean (and SEM) of root-mean-square tractions ($T_{RMS}$), traction maxima ($T_{Max}$), and displacements of neutrophils from healthy donors seeded on 1.5 kPa polyacrylamide gels. Cells were allowed to attach and settle and then imaged before stimulation (“Unstim.”). Cells were then treated with either: (**a**–**c**) 100 ng/ml lipopolysaccharide (“LPS”, n = 34)); (**d**–**f**) a cytokine cocktail of 100 μg/ml IFN-$\gamma$ and 100 ng/ml GM-CSF (“Cyto”, n = 63); or (**g**–**i**) a combined cocktail of LPS, IFN-$\gamma$, and GM-CSF (“L+C”, n = 34). Cells were imaged 30 min post-addition. Displacements were computed in Matlab by T-PT, and displacement heatmaps were generated for three representative cells at 30 min (**c**,**f**,**i**). Tractions were computed using the finite element method in Abaqus. Scale bars are 10 μm, and the white contours represents the boundary of the cell edge. *p < 0.05 (paired Student’s t-test).

Neutrophils were then ex vivo activated with lipopolysaccharide (LPS). As before, each cell was imaged before and after LPS-activation and served as its own unstimulated control. LPS had no statistically significant effect on neutrophil-induced $T_{RMS}$ within 30 min (Fig. 4a), as well as no effect on $T_{Max}$ or displacements (Fig. 4b,c). We next looked at a cytokine cocktail of IFN-$\gamma$ and GM-CSF, which are host-derived pro-inflammatory mediators. Within 30 min, the cytokines decreased $T_{RMS}$, $T_{Max}$, and substrate displacement (Fig. 4d–f). Finally, we looked at a combined cocktail of LPS, IFN-$\gamma$, and GM-CSF, which had no statistically significant effect on $T_{RMS}$, $T_{Max}$, or displacements (Fig. 4g–i). Overall, while neutrophils from septic donors have a greatly increased $T_{Max}$ compared to healthy donors, neutrophils from healthy donors ex vivo activated with LPS and cytokines, not only fail to recapitulate that trend, but even saw reduced tractions by cytokine stimulation.

### Neutrophil traction profiles differ for in-plane and out-of-plane forces

**Figure 5:**
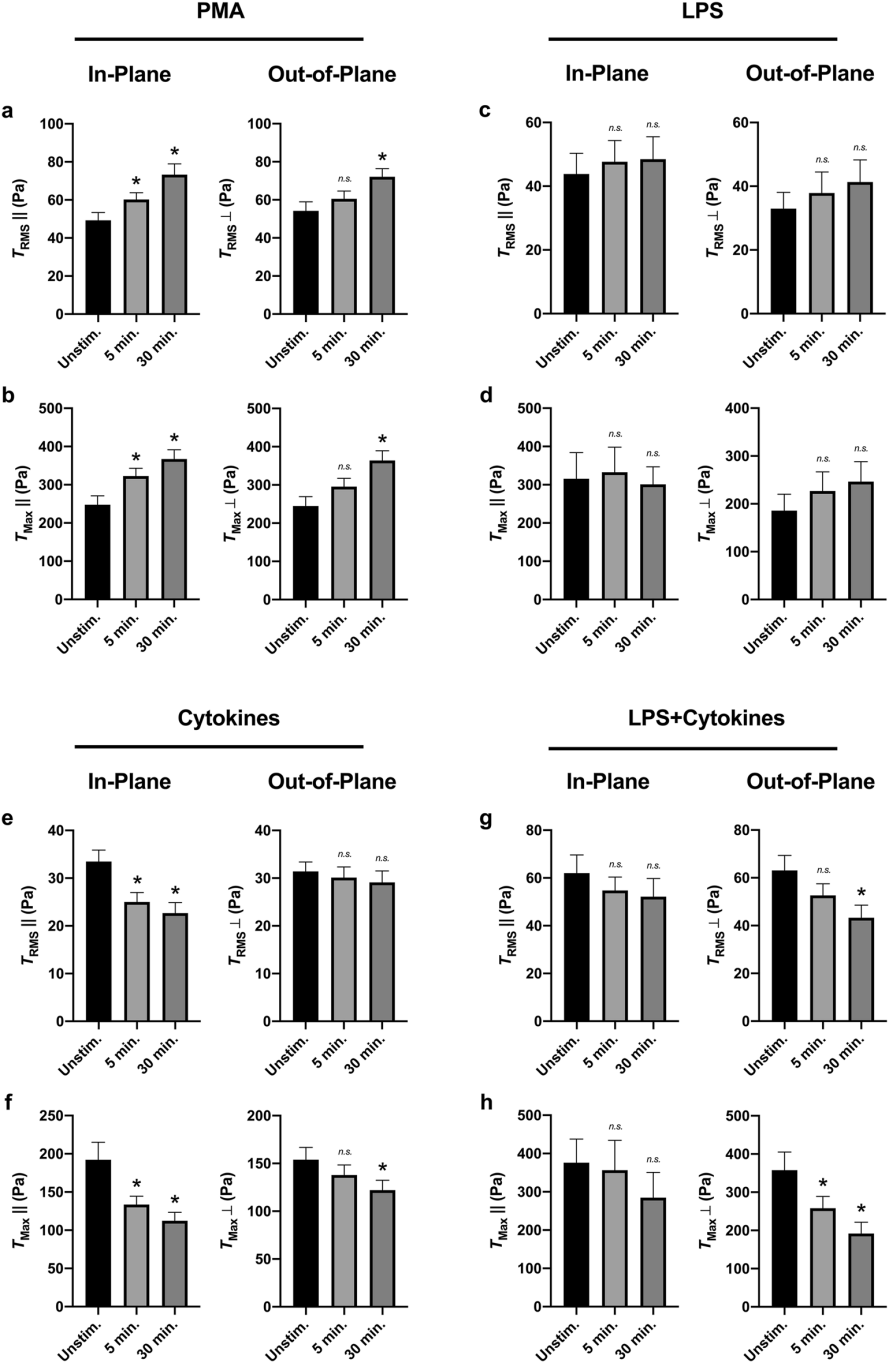
Neutrophils from healthy donors with secondary activation show differential In-Plane ($\parallel$) and Out-of-Plane ($\perp$) tractions. Mean (and SEM) of in-plane/out-of-plane RMS tractions ($T_{RMS}\parallel$/$T_{RMS}\perp$) and in-plane/out-of-plane traction maxima ($T_{Max}\parallel$/$T_{Max}\perp$) of neutrophils from healthy donors seeded on 1.5 kPa polyacrylamide gels. Cells were allowed to attach and settle, and then imaged before stimulation (“Unstim.”). Cells were then treated with either 20nM PMA (n = 70) (**a**,**b**), 100 ng/ml LPS (n = 34) (**c**,**d**), a cytokine cocktail of 100 μg/ml IFN-$\gamma$ and 100 ng/ml GM-CSF (n = 63) (**e**,**f**), or a combined cocktail of LPS, IFN-$\gamma$, and GM-CSF (n = 34) (**g**,**h**). Cells were imaged 5 min post-addition and 30 min post-addition. Tractions were computed using the finite element method in Abaqus. Analyzed using paired Student’s t-test comparing each 5 min timepoint to the unstimulated timepoint and comparing each 30 min timepoint to the unstimulated timepoint, *p < 0.05.

We next sought to determine the role of force directionality during ex vivo activation of neutrophils from healthy donors. Activation by PMA had a comparable effect both on in-plane and out-of-plane $T_{RMS}$ and $T_{Max}$ (Fig. 5a,b), while activation with LPS showed no effect either in-plane or out-of-plane (Fig. 5c,d). However, stimulation by the cytokine cocktail of IFN-$\gamma$ and GM-CSF showed that the majority of the reduction in $T_{RMS}$ and $T_{Max}$ occur in-plane to the substrate (Fig. 5e,f). Conversely, while the cocktail of LPS, IFN-$\gamma$, and GM-CSF had no statistically significant effect on total $T_{RMS}$ and $T_{Max}$ (Fig. 4g–i), when separated into in-plane and out-of-plane components, the cocktail led to a decrease in out-of-plane $T_{RMS}$ and $T_{Max}$ (Fig. 5g,h).

### Differential activation-mediated tractions are mechanosensitive

**Figure 6:**
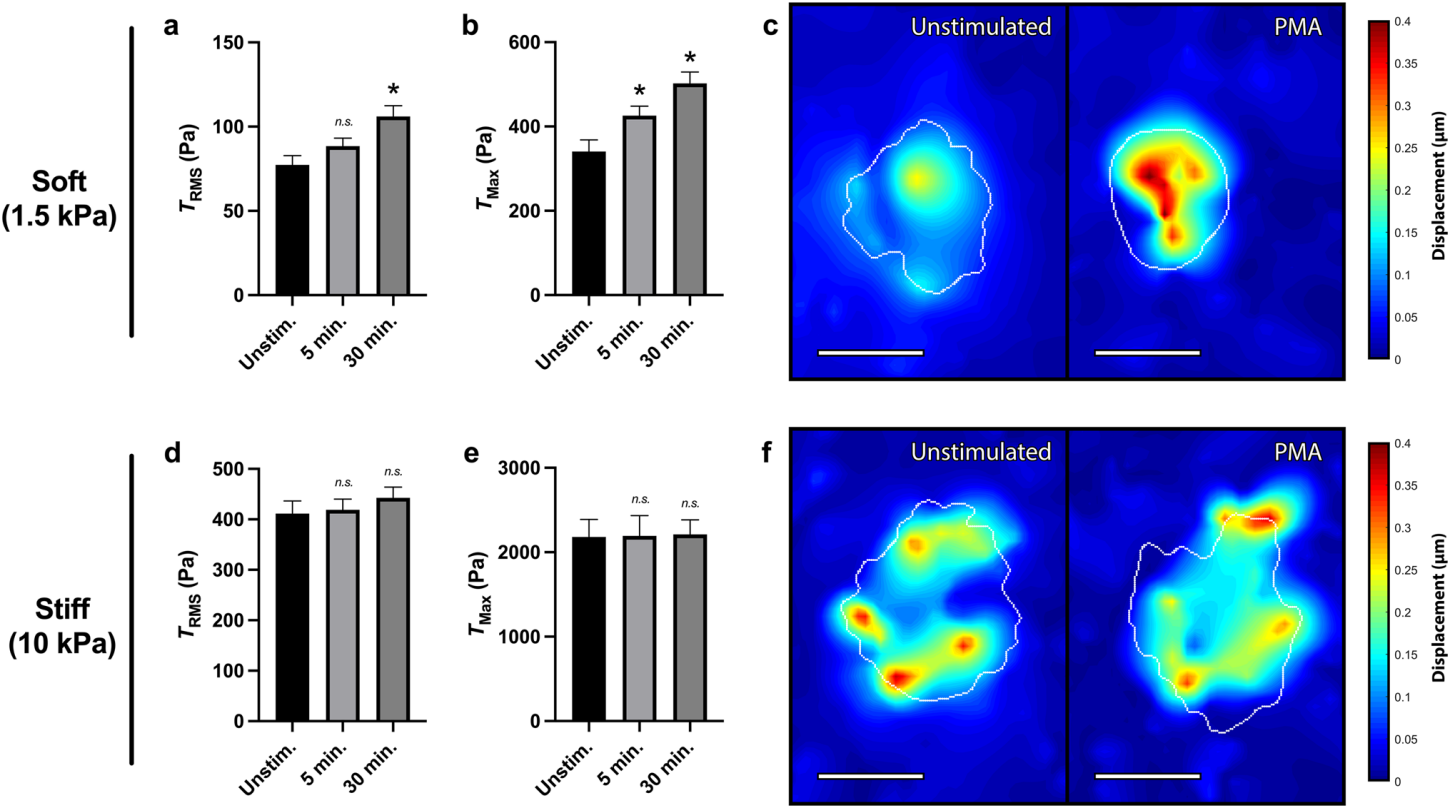
PMA does not increase traction force generation on stiff substrates. Mean (and SEM) of RMS tractions ($T_{RMS}$), traction maxima ($T_{Max}$), and displacements of neutrophils from healthy donors seeded on polyacrylamide gels with a Young’s modulus of either 1.5 kPa (n = 70) (**a**–**c**) or 10 kPa (n = 105) (**d**–**f**). Cells were allowed to attach and settle, and then imaged before stimulation (“Unstim.”). Cells were then treated with 20nM PMA and were imaged 5 min post-addition and 30 min post-addition of PMA. Tractions were computed using the finite element method in Abaqus, and displacement heatmaps were generated for two representative cells at 30 min (**c**,**f**). Scale bars are 10 μm, and the white contours represents the boundary of the cell edge. Analyzed using paired Student’s t-test comparing each 5 min timepoint to the unstimulated timepoint and comparing each 30 min timepoint to the unstimulated timepoint, *p < 0.05.

While previous work has shown neutrophil traction force production to be highly mechanosensitive to underlying substrate stiffness—with unstimulated cells producing greater forces on stiffer substrates—little is known as to how the activation state of the neutrophil effects the stiffness response^14,24^. Therefore, to gain insight into the role of substrate stiffness during force generation, we altered the Young’s modulus of the underlying polyacrylamide gels. While all previous experiments were performed on gels with a Young’s modulus—or stiffness—of 1.5 kPa, we next compared these findings to neutrophils on 10 kPa polyacrylamide gels. Neutrophils activated with PMA generated a statistically significant increase in tractions on 1.5 kPa or “soft” gels (Fig. 6a–c); however, on 10 kPa or “stiff” gels, activation by PMA had no statistically significant effect on neutrophil-generated $T_{RMS}$, $T_{Max}$, or displacements (Fig. 6d–f). Interestingly, the spatial distribution of traction forces across the cell-substrate interface also varied greatly between neutrophils on soft and stiff substrates. While displacement generated by neutrophils on soft substrates—both before and after PMA activation—tended to be localized within the cell center (Fig. 6c), displacements generated by neutrophils on stiff substrates tended to be associated more with the cell edge (Fig. 6f).

**Figure 7:**
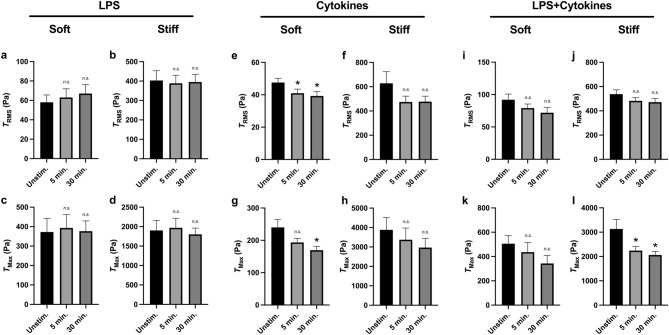
Mechanosensitivity to substrate stiffness is differentially dependent on the activating stimulant. Mean (and SEM) of RMS tractions ($T_{RMS}$) and traction maxima ($T_{Max}$) of neutrophils from healthy donors seeded on polyacrylamide gels with a Young’s modulus of either 1.5 kPa or 10 kPa. Cells were allowed to attach and settle, and then imaged before stimulation (“Unstim.”). Cells were then treated with either: (**a**–**d**) 100 ng/ml LPS (1.5 kPa, n = 34; 10 kPa, n = 30); (**e**–**h**) a cytokine cocktail of 100 μg/ml IFN-$\gamma$ 100 ng/ml GM-CSF (1.5 kPa, n = 63; 10 kPa, n = 52); or (**i**–**l**) a combined cocktail of LPS, IFN-$\gamma$, and GM-CSF (1.5 kPa, n = 34; 10 kP,a n = 52). Cells imaged 5 min post-addition and 30 min post-addition. Tractions were computed using the finite element method in Abaqus. Analyzed using paired Student’s t-test comparing each 5 min timepoint to the unstimulated timepoint and comparing each 30 min timepoint to the unstimulated timepoint, *p < 0.05.

Conversely, neutrophils stimulated with LPS did not generate differential $T_{RMS}$ on either soft (Fig. 7a) or stiff (Fig. 7b) substrates. Ex vivo activation by LPS also did not mediate the differential generation of $T_{Max}$ on either stiffness (Fig. 7c,d). However, neutrophils activated with the cytokine cocktail of IFN-$\gamma$ and GM-CSF generated decreased $T_{RMS}$ on soft substrates (Fig. 7e) but had no statistically significant effect on stiff substrates (Fig. 7f); similarly, $T_{Max}$ of neutrophils with the cytokine cocktail was decreased on soft but not on stiff substrates (Fig. 7g,h). Finally, while the combined cocktail of LPS, IFN-$\gamma$, and GM-CSF had no effect on neutrophil-generated $T_{RMS}$ on either stiffness (Fig. 7i,j) and no effect on $T_{Max}$ on soft substrates (Fig. 7k), the cocktail decreased neutrophil-generated $T_{Max}$ on stiff substrates (Fig. 7l). Overall, these findings indicate that the nature of neutrophil activation differentially regulates mechanosensitivity to substrate elasticity during traction force generation.

### Neutrophil traction force generation as a function of priming

**Figure 8:**
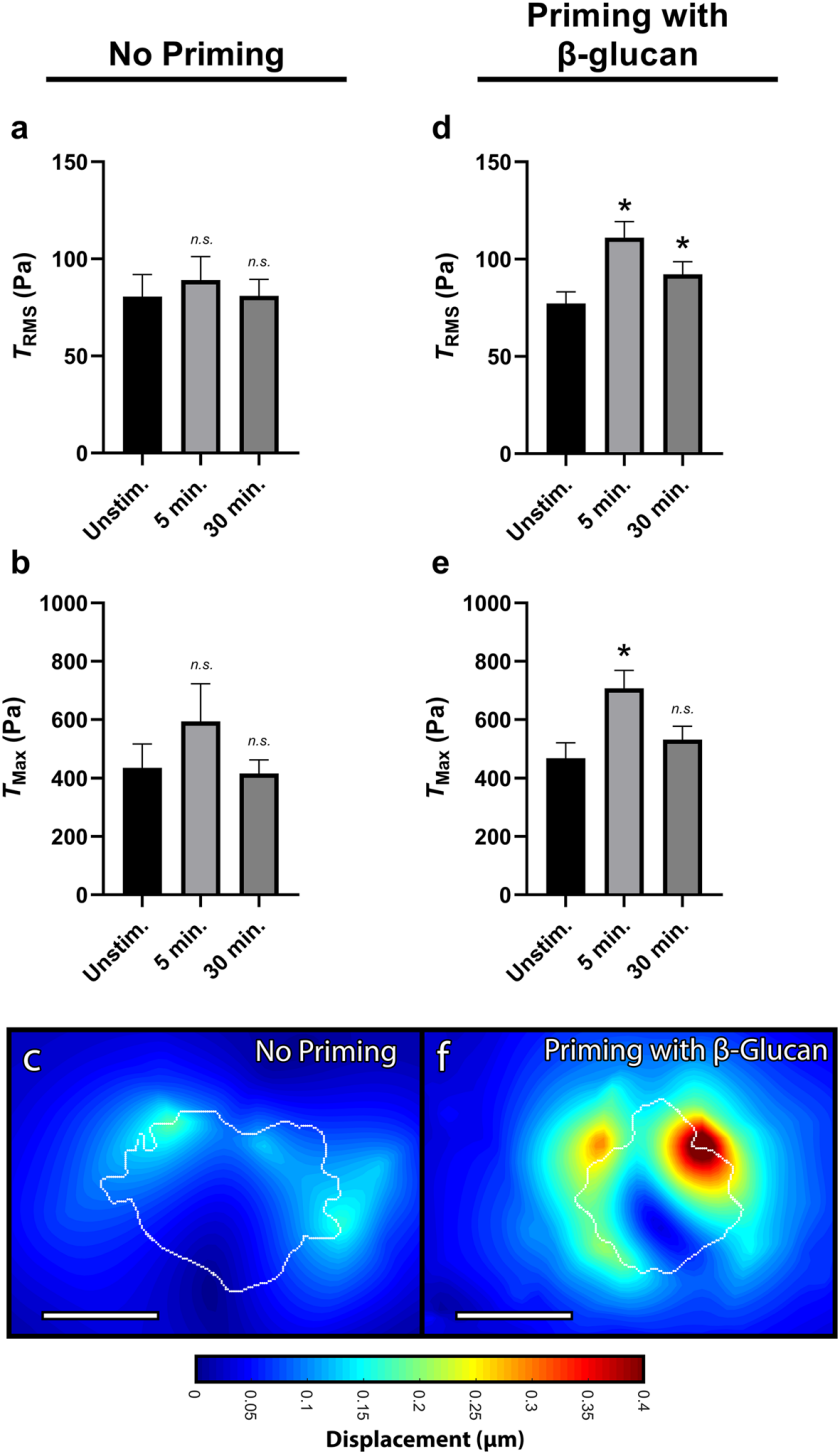
Priming neutrophils with soluble $\beta$-glucan increases RMS traction and traction maxima with fMLF stimulation. Mean (and SEM) of RMS tractions ($T_{RMS}$), traction maxima ($T_{Max}$), and displacements of neutrophils from healthy donors seeded on 1.5 kPa polyacrylamide gels. Cells were allowed to attach and settle, and imaged before the addition of $\beta$-glucan or fMLF (“Unstim.”). (**a**–**c**) Cells with “No Priming” were then treated with 1 μM of fMLF and imaged 5 min and 30 min post-addition (n = 27). (**d**–**f**) Cells with “Priming with $\beta$-glucan” were instead first primed with 20 μg/mL soluble $\beta$-glucan for 10 min and then treated with 1 μM of fMLF and imaged 5 min and 30 min post-fMLF addition (n = 68). Displacements were computed in Matlab by T-PT, and displacement heatmaps were generated for two cells at 5 min post-fMLF (**c**,**f**); tractions were computed using the finite element method in Abaqus. Scale bars are 10 μm, and the white contours represents the boundary of the cell edge. Analyzed using paired Student’s t-test comparing each 5 min timepoint to the unstimulated timepoint and comparing each 30 min timepoint to the unstimulated timepoint, *p < 0.05.

As described above, neutrophil activation refers to a state in which the cell demonstrates augmented functional activity in response to stimulation. However, neutrophils are also capable of being primed which refers to an intermediate state in which changes in neutrophil effector functions are not readily demonstrable but become augmented upon challenge with a second stimulating agent. Prior results from our laboratory have showed that resting peripheral blood neutrophils respond to soluble $\beta$-glucan by achieving a primed state, whereas N-Formyl peptides, such as N-Formylmethionine-leucyl-phenylalanine (fMLF), act as a stimulating agent capable of full-blown cell activation. Therefore, we next sought to determine the effect of fMLF activation on neutrophil-induced traction forces alone as well as in conjunction with priming by $\beta$-glucan to determine if force production is a function of the primed state of the cell. While activation by fMLF alone did not increase $T_{RMS}$ or $T_{Max}$ (Fig. 8a–c), neutrophils that were first primed with soluble $\beta$-glucan and then stimulated with fMLF, had a marked increased in $T_{RMS}$ and $T_{Max}$ immediately following activation (Fig. 8d–f); however, this increase was only transient and dissipated by 30 min. Further, soluble $\beta$-glucan alone did not increase tractions, indicating a non-activating, priming role for traction force generation.

**Figure 9:**
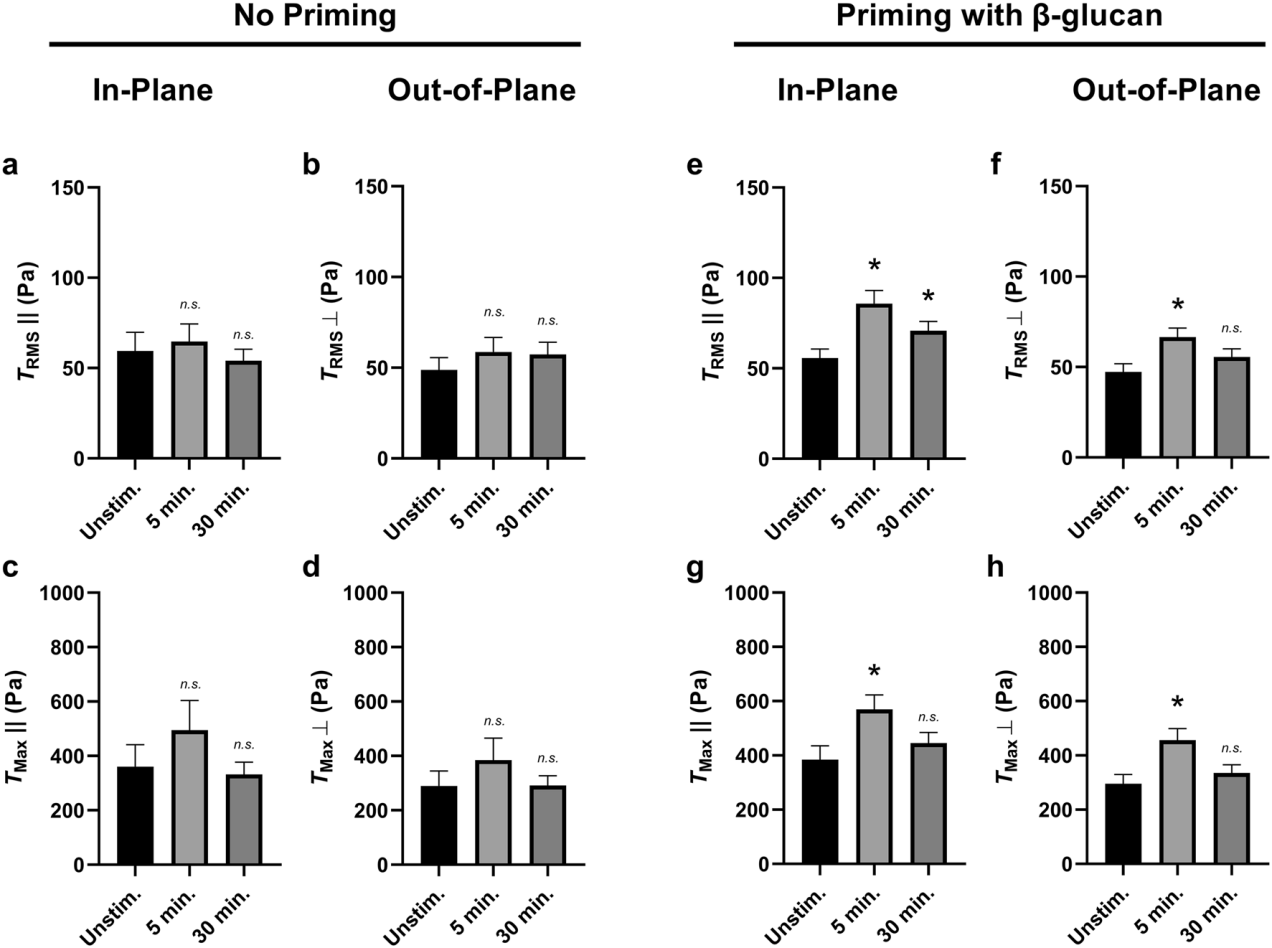
$\beta$-glucan priming increases RMS traction and traction maxima during fMLF stimulation both in-plane and out-of-plane. Mean (and SEM) of in-plane/out-of-plane RMS tractions ($T_{RMS}\parallel$/$T_{RMS}\perp$) and in-plane/out-of-plane traction maxima ($T_{Max}\parallel$/$T_{Max}\perp$) of neutrophils from healthy donors seeded on 1.5 kPa polyacrylamide gels. Cells were allowed to attach and settle and imaged before the addition of $\beta$-glucan or fMLF (“Unstim.”). (**a**–**d**) Cells with “No Priming” were treated with 1 μM of fMLF and imaged 5 min and 30 min post-addition, and the traction quantities (**a**) $T_{RMS}\parallel$, (**b**) $T_{RMS}\perp$, (**c**) $T_{Max}\parallel$, and (**d**) $T_{Max}\perp$ were computed using the finite element method in Abaqus (n = 27). (**e**–**h**) Cells with “Priming with $\beta$-glucan” were first primed with 20 μg/mL soluble $\beta$-glucan for 10 min and then treated with 1 μM of fMLF and imaged 5 min and 30 min post-fMLF addition, and the traction quantities **(e)** $T_{RMS}\parallel$, (**f**) $T_{RMS}\perp$, (**g**) $T_{Max}\parallel$, and (**h**) $T_{Max}\perp$ were computed using the finite element method in Abaqus (n = 68). Analyzed using paired Student’s t-test comparing each 5 min timepoint to the unstimulated timepoint and comparing each 30 min timepoint to the unstimulated timepoint, *p < 0.05.

We next sought to determine the role of traction directionality on this differential priming-mediated force regulation. We first looked at the $T_{RMS}$ of neutrophils activated by fMLF—but without priming by $\beta$-glucan—and found that neither the in-plane nor the out-of-plane components of $T_{RMS}$ showed differential traction generation (Fig. 9a,b). Similarly, the $T_{Max}$ of fMLF-activated but not $\beta$-glucan-primed neutrophils showed no differential traction generation in either the in-plane or out-of-plane components (Fig. 9c,d). We next looked at the $T_{RMS}$ of neutrophils primed by $\beta$-glucan prior to activation by fMLF and found that both the in-plane and the out-of-plane components of $T_{RMS}$ showed increased traction generation (Fig. 9e,f). Similarly, the $T_{Max}$ of $\beta$-glucan-primed and fMLF-activated neutrophils showed a transient increase in traction generation both in the in-plane and the out-of-plane components (Fig. 9g,h). Overall, neutrophils stimulated by fMLF—with no prior priming—show no difference in traction force generation (Fig. 8a–c) even when separating their planar components (Fig. 9a–d). Conversely, the increase in traction generation when neutrophils are primed by $\beta$-glucan prior to activation by fMLF (Fig. 9d–f) seems to be conserved in both planar components (Fig. 9e–h).

**Figure 10:**
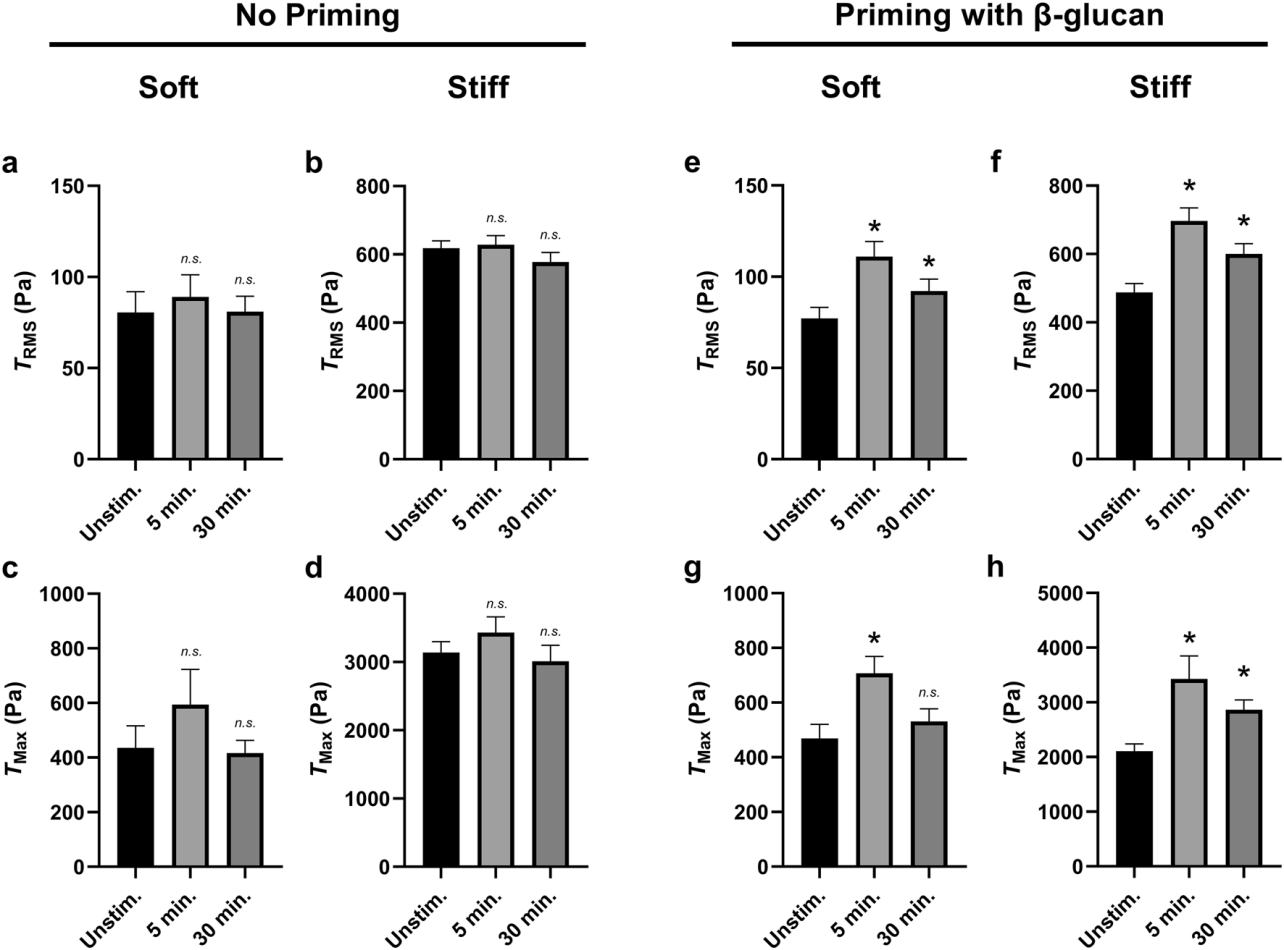
$\beta$-glucan priming increases RMS traction and traction maxima during fMLF stimulation on both soft and stiff substrates. Mean (and SEM) of RMS tractions ($T_{RMS}$) and traction maxima ($T_{Max}$) of neutrophils from healthy donors seeded on polyacrylamide gels with a Young’s modulus of either 1.5 kPa (soft) or 10 kPa (stiff). Cells were allowed to attach and settle and imaged before the addition of $\beta$-glucan or fMLF (“Unstim.”). (**a**–**d**) Cells with “No Priming” were then treated with 1 μM of fMLF and imaged 5 min and 30 min post-addition; the RMS tractions $T_{RMS}$ on 1.5 kPa (**a**) and 10 kPa (**b**) substrates and the traction maxima $T_{Max}$ on 1.5 kPa (**c**) and 10 kPa (**d**) substrates were calculated using the finite element method in Abaqus (1.5 kPa, n = 27; 10 kPa, n = 107). (**e**–**h**) Cells with “Priming with $\beta$-glucan” were first primed with 20 μg/mL soluble $\beta$-glucan for 10 min and then treated with 1 μM of fMLF and imaged 5 min and 30 min post-fMLF addition; the RMS tractions $T_{RMS}$ on 1.5 kPa (**e**) and 10 kPa (**f**) substrates and the traction maxima $T_{Max}$ on 1.5 kPa (**g**) and 10 kPa (**h**) substrates were computed using the finite element method in Abaqus (1.5 kPa, n = 68; 10 kPa, n = 48). Analyzed using paired Student’s t-test comparing each 5 min timepoint to the unstimulated timepoint and comparing each 30 min timepoint to the unstimulated timepoint, *p < 0.05.

We next examined the mechanosensitive role of substrate elasticity on differential traction generation following neutrophil priming. We first looked at the $T_{RMS}$ of neutrophils activated by fMLF—but without priming by $\beta$-glucan; we found that—in addition to soft substrates (Fig. 10a)—neutrophils on stiff substrates did not produce differential $T_{RMS}$ post-fMLF-activation (Fig. 10b). Similarly, activation by fMLF had no effect on $T_{Max}$ on either stiffness (Fig. 10c,d). We next looked at the $T_{RMS}$ of neutrophils primed by $\beta$-glucan prior to activation by fMLF and found that $T_{RMS}$ was increased post-activation on both stiffnesses (Fig. 10e,f). Similarly, neutrophils primed by $\beta$-glucan prior to activation by fMLF generated increased $T_{Max}$ post-activation (Fig. 10g,h).

## Discussion

This study fulfilled two primary objectives. The first was to begin to understand the responsiveness of traction force generation by neutrophils to activating signals generated in vivo by the state of inflammatory disease or in vitro by exposure to priming and/or activating agents. The second objective was to determine the feasibility of applying a newly described means of acquiring traction force data with the use of epifluorescence imaging of cells acquired in a planar field but capable of yielding 3D force mapping. A prior publication from our laboratories describes and validates this technology in detail but used only resting neutrophils to exemplify the method. Here we extend the use of that approach to analyze experimental groups of neutrophils in order to assess the force response of neutrophils to activation stimuli.

TFM allows us to study how neutrophils physically interact with their microenvironment by embedding fluorescent beads into a hydrogel substrate and tracking bead displacement as the cell deforms the gel^14,24,35,36^. Historically, this process was first investigated on 2D imaging modalities, looking at 2D displacement^40–42^. As the technology expanded, so too did the resolution and directionality of these displacements. The next major advance to this system involved the visualization of beads fully embedded throughout the substrate material^35,43,44^. As such, while this technological next-step allowed for the study of 3D displacements, it also required a 3D imaging modality. In addition to requiring a confocal microscope-or other 3D modality-this system also involved high computational costs, which greatly limited its usability and accessibility. However, our laboratory recently developed a novel TFM technique that represents yet another technological step: the visualization of 3D displacements with low computation costs and using only epifluorescence microscopy^38^. Lastly, our novel methodology does not stop at computing 3D displacement; using a finite element method, we are able to convert our 3D displacement measures into 3D traction force measures. While displacement and tractions describe similar mechanical trends when looking within a single set of mechanical properties, displacement is an indirect measure of cellular function and does not take into account the stiffness of the underlying substrates. Therefore, displacement and traction force show very similar trends when comparing only within a single stiffness. However, traction force becomes a necessary readout when comparing between stiffnesses. Therefore, to be able to compare difference stiffnesses (1.5 and 10 kPa), traction force is a necessary readout.

**Table 1:** Summary of activating agents and traction force generation on soft substrates.

Summary of traction force generation			
Activating agent	Effect on traction forces	Differential in/out-of-plane?	Mechanosensitive to stiffness?
PMA	$\Uparrow \Uparrow$	–	Yes
LPS	–	–	–
Cytokines	$\Downarrow$	Yes	Yes
LPS + Cytokines	–	Yes	Yes
fMLF	–	–	–
fMLF + Priming	$\Uparrow$	–	–

*(Left)* Summary of every pharmacological activating agent and its effect on traction forces on soft substrates, *(Center)* and if that effect is differentially regulated in-plane versus out-of-plane to the soft substrate. *(Right)* Summary of every pharmacological activating agent and if it’s aforementioned trend differs on stiff substrate.

The results here show the value of the epifluorescence approach to 3D TFM, which allowed us to identify a regulatory role of the chemical and mechanical microenvironment on neutrophil-generated forces. Moreover, this novel imaging methodology can stratify force generation into in-plane and out-of-plane parameters to compliment the effect of the activation state of the neutrophil, thereby allowing for a greater depth of understanding on how force regulation takes place, summarized in Table 1. PMA is a nonphysiological but potent activator of neutrophils. Known to directly activate PKC, PMA is commonly used as a “gold-standard” or benchmark for neutrophil activation^45^. Its use in Fig. 3 was to provide *in vitro* activation on which the utility of the epifluorescence technique can be clearly determined. Indeed, clear differences in root-mean-square tractions and traction maxima show the direct result of neutrophil activation on traction force generation.

The overarching experimental design used in this study was to survey several priming and activating conditions to provide preliminary indicators as to whether activation is regulatory of force generation and, if so, if force generation is differentially regulated by the nature of the activating or priming stimuli. One finding that supports differential force generation as a function of activation conditions was that neutrophils from septic donors have increased traction forces as compared to healthy donors, while neutrophils from healthy donors ex vivo activated with pro-inflammatory cytokines actually generated decreased tractions as compared to untreated.

Sepsis is a systemic inflammatory response to a source of infection, and this disease state can lead to excessive and unbridled neutrophil activation due to the release of a storm of host derived cytokines and microbially-derived molecules^6,8–12^. Neutrophils from septic patients had comparable root-mean-square displacements and root-mean-square tractions as neutrophils from healthy donors; however, their total forces were far greater. This indicates that most of the increase in septic neutrophil forces was likely due to an increase in spread area; when normalizing for cell area—such as with $T_{RMS}$—the differences were negligible. Neutrophils from septic patients were additionally highly dysregulated in traction maxima, indicating that their highest single peaks were much higher than seen in neutrophils from healthy donors. Finally, the spatial distribution of neutrophil-generated displacements and tractions—visualized with the displacement $|{\textbf {u}}|$ and traction $|{\textbf {t}}|$ magnitude heatmaps—indicate that neutrophils from septic donors have a far more irregular and punctate traction pattern. Overall, neutrophils from septic donors seem to be highly dysregulated both in spread areas—leading to an increase in total forces—in their traction maxima, and in a lack of traction field uniformity—as seen in their displacement $|{\textbf {u}}|$ and traction $|{\textbf {t}}|$ magnitude heatmaps. It would therefore suggest that force dysregulation is a component of the effect of sepsis on the neutrophil. It would be of interest to determine if force dysregulation tracks with the clinical course and severity of disease and whether other critical injuries such as trauma and burn similarly affect force which could in turn underlie the effect of disease on motility and anti-microbial activities.

While the traction profile of neutrophils from septic donors might indicate that increased cellular activation increases adhesion, contractility, and—by extension—traction force generation, we found that not all agonists are equal. While maximally activating neutrophils from healthy donors with PMA—which is receptor independent^45^—had the expected results of increased adhesion and therefore increased tractions (Fig. 3), examination of common receptor agonists associated with sepsis had profoundly divergent results (Fig. 4). Neutrophils from healthy donors ex vivo activated by LPS—a well known TLR4 agonist^46^—had no change in their traction profile. Further, neutrophils co-activated by a combination of IFN-$\gamma$ and GM-CSF actually had reduced $T_{RMS}$ and $T_{Max}$, indicating fewer and smoother cell-generated tractions. IFN-$\gamma$ is a host-derived neutrophil-activating cytokine that is often associated with systemic inflammation and which has been implicated in both pro- and anti-inflammatory deleterious roles^47–50^, and GM-CSF is a myelopoietic growth factor associated with neutrophil maturation and priming^48,51^. Further, these differential traction profiles for LPS-alone, the two-way combination of IFN-$\gamma$ and GM-CSF, and the three-way combination of LPS, IFN-$\gamma$, and GM-CSF were mechanically sensitive to direction and differentially regulated their in-plane and out-of-plane traction profiles (Fig. 7).

In addition to a state of maximal activation, neutrophils can also achieve a state of partial, or intermediate, activation referred to as priming^52^. In this state, the cell can undergo adhesion to the substrate and mobilize intracellular signaling functions but cannot elicit a major functional effect unless triggered with a second stimulant^53,54^. The soluble $\beta$-glucan utilized in Figs. 8, 9, 10 at the concentrations employed has been shown to induce this primed state^55,56^. This is so because soluble $\beta$-glucan can stabilize the $\beta$2 integrin in an intermediate molecular conformation and also induce phosphorylation events indicative of a cellular response; soluble $\beta$-glucan by itself does not induce the active conformer of CR3 or the respiratory burst^55^. Results shown demonstrate that 1 μM fMLF alone had no effect on either $T_{RMS}$ or $T_{Max}$ generated by adherent neutrophils (Figs. 8, 9, 10). However, after priming with soluble $\beta$-glucan, fMLF showed a statistically significant effect on both traction measures. Further, the increased traction forces generated by this two-hit model of $\beta$-glucan priming followed by fMLF activation were conserved in both directional dimensions and on both stiffnesses. Interestingly, this makes $\beta$-glucan priming followed by fMLF activation the only priming/activating agents examined within this study that increased traction forces on stiff substrates. These findings, while preliminary, suggest that neutrophil-generated traction forces are subject to the effects of priming followed by activation.

While this study looked at a multitude of differential neutrophil activation modulators, this was by no means an exhaustive list. Adapted from historically used neutrophil ex vivo stimuli, these chosen activation agents engaged only a short list of the receptors neutrophils utilize during inflammation and injury. To summarize, LPS is a bacterial PAMP and common TLR4 agonist used in neutrophil research; fMLF is a common formyl peptide receptor 1 (FPR1) agonist and which derivatives of can be both pathogen-derived and host-derived; IFN-$\gamma$ (engaged by IFN-$\gamma$R1) and GM-CSF (engaged by GM-CSFR) are both host-derived cytokines with varying roles in pro- and anti-inflammatory regulation; $\beta$-glucan is a component of the fungal cell wall and is an agonist for the lectin-like site of CR3; and PMA is a receptor-independent activating agent. Additionally, while not all receptor-mediating activating agents have identical effects on traction generation when studied individually, these effects are further changed when agents are used in combination. We found differential combinatorial effects both with regards to activating agents—as seen with LPS and inflammatory cytokines alone and in conjunction—as well as with priming agents—as seen with fMLP with and without priming. Given that neutrophils are bombarded by a number of activating signals during diseases of inflammation, a better understanding of how mechanical and humoral properties of the microenvironment coalesce to fine-tune production of traction force must consider these multivariant components alone as well as together.

Taken together, these represent only a small fraction of mediators of the neutrophil activation state, and one future direction might be the expansion of this list. Of particular note, it would be of interest to look at a TLR2 agonist, such as Pam3CSK, as well as a more comprehensive list of sepsis-associated pro-inflammatory cytokines, such as IL-1$\beta$, IL-6, IL-12, and TNF-$\alpha$^57^; another interesting activating agent might also include serum from a septic donor. Additionally, the IFN-$\gamma$ and GM-CSF traction force phenotype was notable in that it reduced neutrophil-generated tractions. However, the two cytokines were only ever used in tandem. Therefore, in addition to expanding the list of studied cytokines, it would also be of interest to examine the individual roles of IFN-$\gamma$ and GM-CSF. Moreover, each condition could be greatly expanded to include variables of dose, time, and use of stimulants in combination—as occurs during inflammation—to better understand the force response of the neutrophil.

Lastly, another major advancement of the activation-mediated differentially-mechanosensitive traction force studies would be the inclusion of motility and migration. While multiple metrics to evaluate displacements, tractions, and forces were utilized, this is only a partial description of what mechanical processes the cell is undergoing. As such, some next steps might include chemokinesis and chemotactic assays on soft and stiff hydrogels with an array of stimulants and/or chemoattractants. Metrics for motility could include speed, directionality, persistence, and mean-squared-displacement (MSD). This is of particular note because neutrophil motility metrics have been shown to be highly mechanosensitive to stiffness^14^. However, how the activation state of the neutrophil regulates this mechanosensitivity is unknown and would be a beneficial companion study to the traction force metrics.

In summary, we show that mechanosensitive neutrophil-generated traction forces are highly regulated by the activation state of the cell, as well as on the nature of the activating signals. By identifying key points of mechano-dysregulation via aberrant traction force machinery within the neutrophil, the field of mechanobiology could offer new therapeutic targets for gaining control of the septic neutrophil. Therefore, moving forward, we find that the study of critical illness must not only consider the biochemical component of cellular function but also further bridge the gap between the mechanical and the biochemical functions. With a disease as highly complex as sepsis, only with the most complete picture can we begin to fully understand it.

## Methods

### Polyacrylamide gel preparation

Gel substrates were prepared using a procedure adapted from Oakes et al.^14^ and polymerized between two glass 25 mm coverslips. The bottom coverslip was functionalized with 0.5% 3-Aminopropyltriethoxysilane (Sigma 281778) in ethanol followed by 0.5% glutaraldehyde (Polyscience 07710-100) in deionized water for hydrophilicity. The top coverslip was coated with 0.1% Poly-l-lysine for 1 h and then blown dry; it was then coated with a 1:100 dilution in deionized water of suspended RFP-fluorescent 0.5 μm carboxylate-modified polystyrene beads for 1 h and then blown dry. Polyacrylamide gel substrates were then prepared using varying concentrations of acrylamide (Bio-Rad) and N,N-methylene-bisacrylamide (Bio-Rad) to achieve either a Young’s modulus of either 1.5 kPa or 10 kPa; 1.5 kPa substrates were made with 3% acrylamide and 0.2% bisacrylamide, and 10 kPa substrates with 5.2% acrylamide and 0.19% bisacrylamide. Polyacrylamide solutions were vortexed and then polymerized through the addition of excess tetramethylethylenediamine (Sigma T9284) and ammonium persulfate (Sigma A3678). Immediately after the addition of these polymerizing agents, 15 μL of gel solution was added to the hydrophilic bottom coverslip and then flattened by the bead-coated top coverslip. The gel solution was allowed to polymerize at room temperature for 15 min and then soaked in water for 1 hour to remove any unpolymerized acrylamide, after which the top coverslip was removed while the single layer of beads remained embedded in the gel. As previously described by Oakes et al.^14^, gel substrates were then coated with human fibronectin (Gibco 33016015) using the photoactivatable crosslinker sulfo-SANPAH (Sigma 803332). A 1 mg/ml solution of sulfo-SANPAH was added to the gels in the dark for 10 min at room temperature and allowed to covalently bind to the acrylamide. Gels were then placed under ultraviolet light for 15 min to crosslink the sulfo-SANPAH to the gel. After cross-linking, excess sulfo-SANPAH solution was aspirated off and replaced by fresh sulfo-SANPAH and then immediately placed under ultraviolet light again for 15 min. The gels were then washed four times with water, and fibronectin (BD Biosciences 356008) was added at 200 μg/ml in 50 mM pH 7.0 HEPES. The fibronectin was then allowed to crosslink overnight at 4 °C. Finally, prior to the experiment, gels were washed three times in Liebovitz L-15 media to remove any excess fibronectin. The elasticity of the gels has been shown to be unaffected by the protein coating procedure, and the density of protein on the surface of the gel is unaffected by the elasticity of the gels^14^.

### Septic patient enrollment

Study approval was obtained from the Institutional Review Board of Rhode Island Hospital. Written informed consent to participate was provided by the patients or their surrogates. Critically ill septic patients were prospectively enrolled from the surgical ICU and the trauma ICU. Septic patients were identified as those with two or more systemic inflammatory response syndrome criteria with a source of infection. Patients were diagnosed with sepsis based on clinical evidence or microbiological data. Patients were enrolled within 48 hours of their diagnosis or admission. The patient charts were reviewed for demographics, laboratory values, and vitals. In addition, charts and microbiologic data were reviewed to identify the source of sepsis. Data collected was used to calculate Sequential Organ Failure Assessment (SOFA) score at time of blood draw. Patients were enrolled if they were 18 years of age or older and were excluded if they had a massive blood transfusion of 4 or more units.

### Healthy donor enrollment

Healthy volunteers were defined as subjects having no known chronic systemic, oral diseases, or acute infection during the past one month. Smokers were excluded since smoking may affect neutrophil activation. Venous human blood was collected solely for the isolation of neutrophils for use in our *in vitro* functional assays. Blood samples were collected anonymously and involved the withdrawal of 10–30 ml of blood by venipuncture of normal human volunteers. Blood was drawn no more than once a week from normal laboratory volunteers of at least 110 lbs in weight and did not exceed 550 cc in any eight week period. Blood was not obtained from minors, pregnant women, prisoners, mentally retarded or mentally disabled patients or volunteers. Further, blood was not obtained from any normal volunteers with concomitant acute infectious processes.

### Isolation of human neutrophils

As stated above, whole blood was obtained from healthy or septic donors with written informed consent and in accordance with the guidelines from and approval from the Rhode Island Hospital Institutional Review Board. Blood was collected in EDTA-containing Vacutainer tubes and separated by Histopaque-1077, followed by a sedimentation step with 3% 400–500 kDa Dextran. Contaminating erythrocytes were then removed via hypotonic lysis, and neutrophils were resuspended in cation-free HBSS.

### Ex vivo activation of human neutrophils

The fibronectin-coated gel coverslip was placed in a 25 mm coverslip holder, and 1 mL of Leibovitz L-15 was added. 50,000 neutrophils were then added and allowed to settle and attach to the gel for 15 min. After attachment, 20–60 adherent cells were then selected and imaged in both RFP and Brightfield for the unstimulated timepoint. After the initial timepoint was imaged, the ex vivo stimulant was then added: lipopolysaccharide (LPS) at 100 ng/mL, N-Formylmethionine-leucyl-phenylalanine (fMLF) at 1 μM, Phorbol 12-myristate 13-acetate (PMA) at 20nM, soluble $\beta$-glucan at 20 μg/mL, interferon-$\gamma$ (IFN-$\gamma$) at 100 μg/mL, and/or granulocyte-macrophage colony-stimulating factor (GM-CSF) at 100 ng/mL. For treatment groups without $\beta$-glucan-priming, the stimulants were as follows: LPS alone; cytokines alone (IFN-$\gamma$ and GM-CSF); LPS and Cytokines (IFN-$\gamma$ and GM-CSF); fMLF alone; and PMA alone. After the stimulant was added, the selected neutrophils were imaged 5 min post-addition and 30 min post-addition.

For the treatment group with $\beta$-glucan priming, the selected neutrophils were imaged prior to the addition of any treatment for the unstimulated timepoint. Soluble $\beta$-glucan (HiberCell, Inc, Roseville, MN) was then added, and the same cells were imaged 10 min post-addition. After the $\beta$-glucan timepoint, fMLF was then added, and the selected neutrophils were imaged 5 min post-addition and 30 min post-addition. After the image acquisition of all stimulant timepoints, the cells were then removed from the gels by the addition of 2.5% sodium dodecyl sulfate (SDS), and a final image of each position was taken as the reference condition.

### Live-cell imaging parameters and cell selection

All experiments were imaged on a Nikon TI-2 epifluorescent microscope using a 40x air objective with a 0.6 numerical aperture. Additionally, all experiments were done with an aligned correction collar to account for the air/glass/water interfaces. An Okolab enclosure around the TI-2 maintained the apparatus at $37\;^{\circ }\text {C}$ and 5% $\text{CO}_{2}$ for the duration of the experiments. After initial cell selection, a three-dimensional stack of epifluorescence images were then taken centered on the beads. These volumetric images had an XY μm-per-pixel ratio of 0.16 and had a Z-step size of 0.3 μm, with a typical experiment imaging 100 slices.

Only cells adherent to the underlying substrate were selected for imaging. The *n* represents the number of individual neutrophils imaged and analyzed, with an *N* > 3 for individual septic or healthy donors. Cell masks were generated by manually outlining the cell boundary and converted to white outlines in the contour plots.

### Cell-induced displacements and finite element mesh

To determine the traction applied to the substrate by a cell in a given experiment, we utilized finite-element calculations in Abaqus/Standard to solve a boundary-value problem in which the measured displacements from topology-based particle tracking (T-PT) were applied to the surface of a substrate. The substrate was modeled as a square-cross-section block with a side length and height of 0.192 mm and 0.024 mm respectively. The block was intended to approximate an infinite half space, and the dimensions were selected to be large enough so that these values did not affect the calculated tractions. The finite-element mesh for the substrate was made up of 148,000 Abaqus-C3D8H elements (eight-node, three-dimensional, hexahedral, constant-pressure elements). The polyacrylamide substrate was modeled as a hyperelastic, nearly-incompressible neo-Hookean material with ground-state shear modulus G and ground-state bulk modulus K. For the 1.5 kPa gel, the ground-state shear and bulk moduli were G = 0.517 kPa and K = 5 kPa; for the 10 kPa gel, the ground-state shear and bulk moduli were G = 3.45 kPa and K = 33.3 kPa.

The measured displacements were only mapped to select nodes on the surface of the substrate within a square region in the neighborhood of the cell, further discussed below. The mesh on the surface contained a square patch with a refined uniform mesh made up of 10,000 element faces centered on the substrate surface. The side length of the square patch was 0.048 mm, which was larger than the spread length of any given cell. Outside the square patch, the mesh resolution was gradually coarsened with the distance from the center of the substrate, and the top surface outside the square patch was traction free. For the remaining boundary conditions, all displacement components were prescribed to be zero for the nodes on the bottom of the block such that *u*$_x$ = 0, *u*$_y$ = 0, *u*$_z$ = 0, where *x* and *y* denote the in-plane directions, and *z* is the out-of-plane direction to the substrate surface, and the four side surfaces of the block were traction free.

For the selected nodes within the square patch on the top surface, displacement boundary conditions were extracted from the topology-based particle tracking data. The displacement data from T-PT was discrete in nature, consisting of one displacement vector per bead embedded on the substrate. To match the bead positions with the nodal coordinates in the finite-element mesh, the displacement data underwent pre-processing. First, the surface displacement field within the neighborhood of the cell was interpolated based on the bead displacement data using the Matlab function “ScatteredInterpolant,” and contour plots of the displacement components were generated and overlayed with an outline of the estimated boundary of the cell. In some cases, high-magnitude out-of-plane displacements (*u*$_z$) occured at isolated positions outside of the cell and were filtered out. The cell-induced displacements corresponding to the beads were then used to re-interpolate the displacement field and generate updated contour plots of the displacement components. The updated plots were then compared with the original plots to ensure that the filtering process did not affect the displacement field within the region of the cell and its immediate vicinity.

We next identified the center of the region in which the interpolated displacement field was mapped onto the nodes of the square patch of the substrate surface mesh. Due to high variability in cell morphologies, local displacement peaks were first located based on the displacement magnitude and its spatial gradient. Then, the center of the mapped region was determined by averaging the Cartesian coordinates of the locations of the local peaks.

The next step was to determine the selected surface nodes on which displacements would be applied. The selection process was based on a threshold magnitude for the interpolated displacements at pixel locations within an image. An image containing a single cell was 400 × 400 pixels (i.e., 64 μm × 64 μm), and therefore significantly larger than the cell. The displacement magnitude threshold was defined as the average displacement magnitude over all pixels within an image plus one standard deviation. Above this threshold, the displacement in the vicinity of cell was treated as physically-meaningful displacement generated by the cell. Taking the mapping center as the origin of a 2D polar coordinate system, the largest radius from the center to a pixel with a displacement magnitude just above the threshold defined a circle, which was then discretized into 720 evenly spaced points around the circumference with one point per 0.5°. The displacement magnitudes at these points were calculated from the interpolated displacement field. If the magnitude was lower than the threshold, the radius of the point was gradually reduced by small increments until the magnitude reached the threshold and increased when the magnitude was larger than the threshold. Regions of low-level noise having displacement magnitudes above the threshold were manually identified and jumped over, so that the radius of a point could be further reduced. Once this process was completed, the 720 points enclosed the region of physically-meaningful displacement generated by the cell. The displacement field was then mapped onto selected nodes of the finite element mesh with the mapping center coinciding with the central node of the surface mesh. The nodes within the area enclosed by the points were assigned a displacement boundary condition with values calculated from the interpolated displacement field. The element faces inside the refined mesh region but outside the enclosed region therefore remained traction free.

### Finite element simulations for traction measures

The boundary-value problem was then solved using a large-deformation, static analysis step in Abaqus/Standard. For each simulation, the reaction force vectors ***F***$_m$ at each node *m* on which the displacement was specified were extracted using a Python script. From the extracted data, the nodal traction vectors ***t***$_m$ were calculated as ***t***$_m$ = ***F***$_m$/*A*$_m$ where *A*$_m$ was the undeformed area corresponding to node *m*. Since the surface mesh in the square region in the neighborhood of the cell consisted of equally sized squares, *A*$_m$ was equal for all nodes. Nodal reaction force vectors were decomposed into an in-plane reaction force vector ***F***$_m^{\parallel }$ = (*F*$_m^{x}$, *F*$_m^{y}$) and an out-of-plane reaction force component *F*$_m^{z}$. Likewise, traction vectors were decomposed into an in-plane traction vector ***t***$_m^{\parallel }$ = (*t*$_m^{x}$, *t*$_m^{y}$) and an out-of-plane traction component *t*$_m^{z}$. As a measure of the forces applied by a cell on the substrate, we utilized the total force^24^:

1 $$\begin{aligned} F = \int _{A}^{} |{{\varvec{t}}}| \,\, da , \end{aligned}$$

where integration was performed over the undeformed geometry, in which *A* was the spread area of the cell in the reference configuration. The total force is affected by both the tractions applied by the cell and its spread area. To remove the effect of spread area and quantify the traction fluctuation field induced by the cell, we defined the root-mean-square (RMS) traction as

2 $$\begin{aligned} T_{RMS} = \left( \frac{1}{A} \int _{A}^{} {{\varvec{t}}} \cdot {{\varvec{t}}} \,\, da \right) ^{\frac{1}{2}}. \end{aligned}$$

For our discrete data, the total force and RMS traction were

3 $$\begin{aligned} F = \sum _{m=1}^{M} \left( {{\varvec{F}}}_{m} \cdot {{\varvec{F}}}_{m}\right) ^{\frac{1}{2}}\qquad \text {and}\qquad T_{RMS} = \left( \frac{1}{M} \sum _{m=1}^{M} {{\varvec{t}}}_{m} \cdot {{\varvec{t}}}_{m} \right) ^{\frac{1}{2}}, \end{aligned}$$

where *M* was the total number of nodes on which the displacement was specified. Further, the in-plane total force and the out-of-plane total force were defined as follows:

4 $$\begin{aligned} F_{\parallel } = \sum _{m=1}^{M} ({{\varvec{F}}}_{m}^{\parallel }\cdot {{\varvec{F}}}_{m}^{\parallel })^{\frac{1}{2}} \qquad \text {and}\qquad F_{\perp } = \sum _{m=1}^{M} |\textit{F}_{m}^{z}|. \end{aligned}$$

Similarly, we defined the in-plane RMS traction and out-of-plane RMS traction as follows:

5 $$\begin{aligned} T_{RMS}^{\parallel } = \left( \frac{1}{M} \sum _{m=1}^{M} {{\varvec{t}}}_{m}^{\parallel } \cdot {{\varvec{t}}}_{m}^{\parallel } \right) ^{\frac{1}{2}}\qquad \text {and}\qquad T_{RMS}^{\perp } = \left( \frac{1}{M} \sum _{m=1}^{M} (\textit{t}_{m}^{z})^{2} \right) ^{\frac{1}{2}}. \end{aligned}$$

Finally, we defined the maximum traction, maximum in-plane traction, and maximum out-of-plane traction as follows:

6 $$\begin{aligned} T_{Max} = \max _{m} | {{\varvec{t}}}_{m}|, \qquad T_{Max}^{\parallel } = \max _{m} | {{\varvec{t}}}_{m}^{\parallel }|, \qquad \text {and}\qquad T_{Max}^{\perp } = \max _{m} | t_{m}^{z}|. \end{aligned}$$

### Global force and moment balance

To verify that all of our measurements are in static equilibrium, we compute the sum of all forces and moments acting on each cell to verify that static force and moment equilibrium are satisfied. The overall procedure is similar to our previous methodology^44,58^. Traction distributions acting on each cell are calculated using finite element simulations which enforce equilibrium. Therefore, forces are balanced at every node within the force tolerance of the nonlinear finite-element solver, which is on the order of 10^−17^–10^−15^ N for the 1.5 kPa gels and on the order of 10^−17^–10^−14^ N for the 10 kPa gels, respectively, which is consistent with our determined experimental noise floor, and similar to our previously reported values^44,58^.

### Resolution and measurement sensitivity

Since the determination of the surface tractions involves calculations of material strains, the sensitivity of both our displacement and traction measurements needed to be assessed. This was accomplished by computing the displacement and surface traction fields in areas identified to be devoid of any cells. Using standard statistical error analysis, we determined that we can accurately resolve any displacements and tractions greater than 0.0702 μm and 8.9353 Pa for all samples with a Young’s modulus of 1.5 kPa, and any displacements and tractions greater than 0.0696 μm and 13.1427 Pa for all samples with a Young’s modulus of 10 kPa. Similar analyses of the maximum resolution sensitivity for 2D and 3D TFM methodologies can be found elsewhere^44,58–60^.

## Supplementary Information

Supplementary Figures.

## Data Availability

All data and analyses supporting the findings of this study are available from the lead contact upon reasonable request.
