# Supplementary material for: Mechanosensitive traction force generation is regulated by the neutrophil activation state
Source: Sci Rep. 2023 Jul 9;13:11098. doi: 10.1038/s41598-023-37997-y (PMC10330213; doi:10.1038/s41598-023-37997-y)
Supplement: Supplementary file 1 — Supplementary Figures. [file 41598_2023_37997_MOESM1_ESM.pdf]

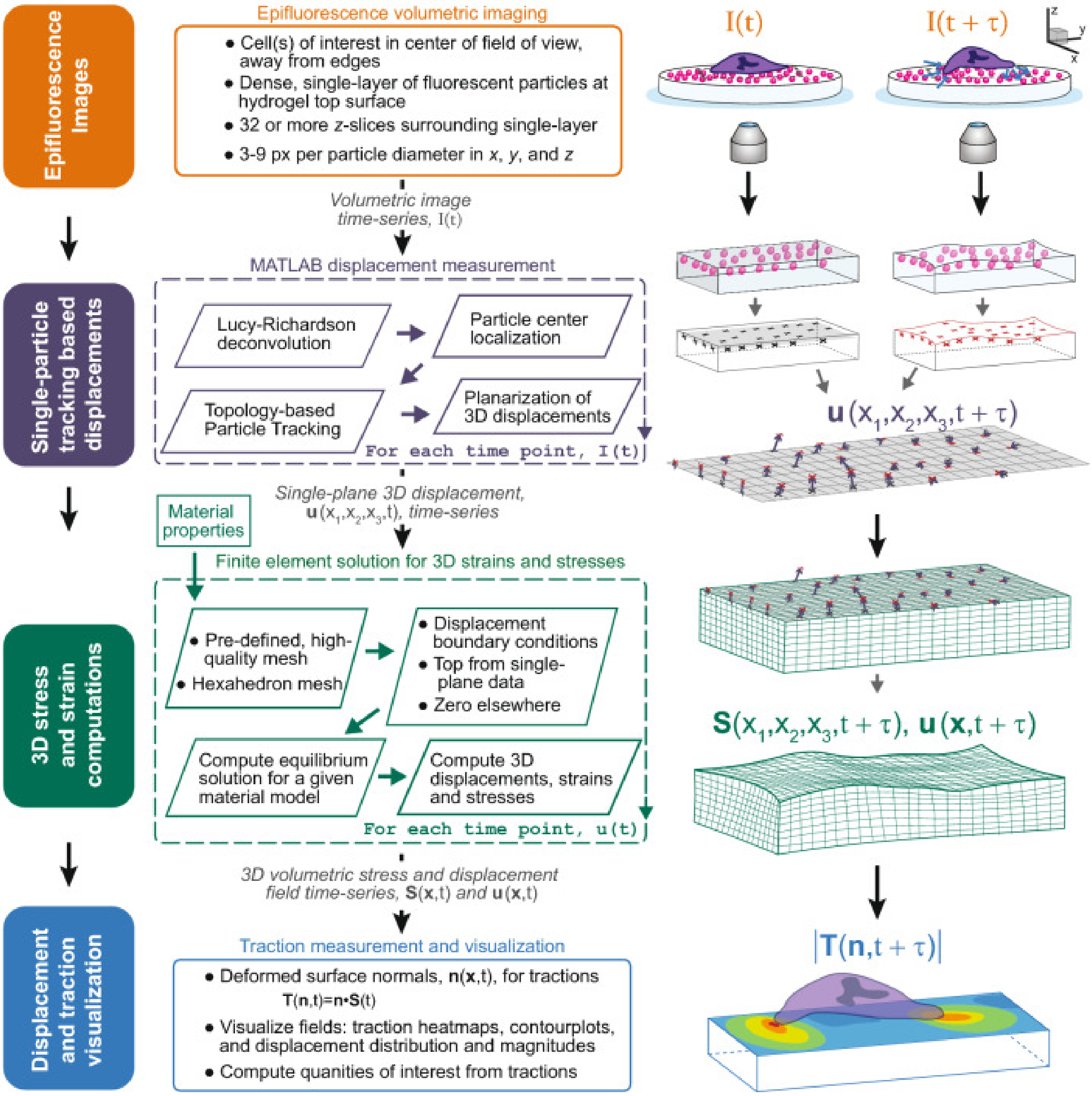

Figure 1. **Representative workflow for our traction force methodology.** Our previously published workflow for converting volumetric epifluorescence images into 3D traction forces. **(Orange)** Volumetric images of the fiducial markers (0.5 μ fluorescent beads) are acquired before and after cell activation. **(Purple)** These images then undergo a Lucy-Richardson deconvolution and displacement of the beads between the two timepoints is determined via a Topology-based Particle Tracking (T-PT) method, as described by Patel et. al. 2018. **(Green** The material properties of the substrate (such as the 1.5 kPa or 10 kPa stiffness) are then utilized with the displacement data using a finite element method to compute the strains and stresses. **(Blue)** Lastly, the traction force is calculated and visualized. This figure was previously published in our methods paper by Hazlett et. al. 2020 and has been reproduced here as allowed via the Copyright Clearance Center.

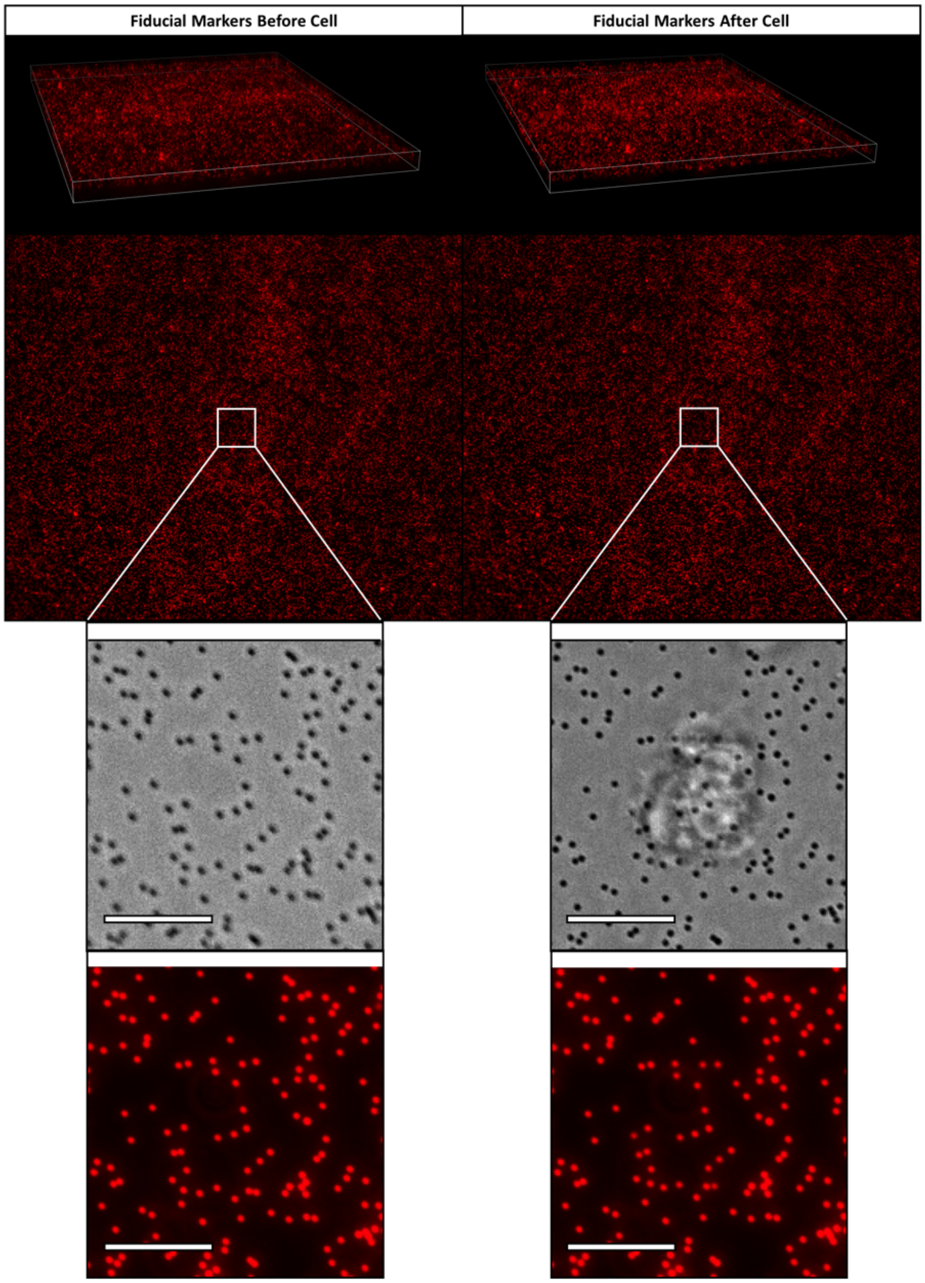

Figure 2. Representative images of fluorescent markers. Representative RFP and brightfield images of a 10 kPa gel with a single layer of 0.5 μm fluorescent beads before **(left)** and after **(right)** the addition of a neutrophil. A single brightfield image at the cell-gel interface was taken and then a volumetric RFP image stack above and below the cell-gel interface was taken prior to the addition of cells and 15 minutes after the addition of cells to allow for cell adhesion. Scale bars are 10 μm.

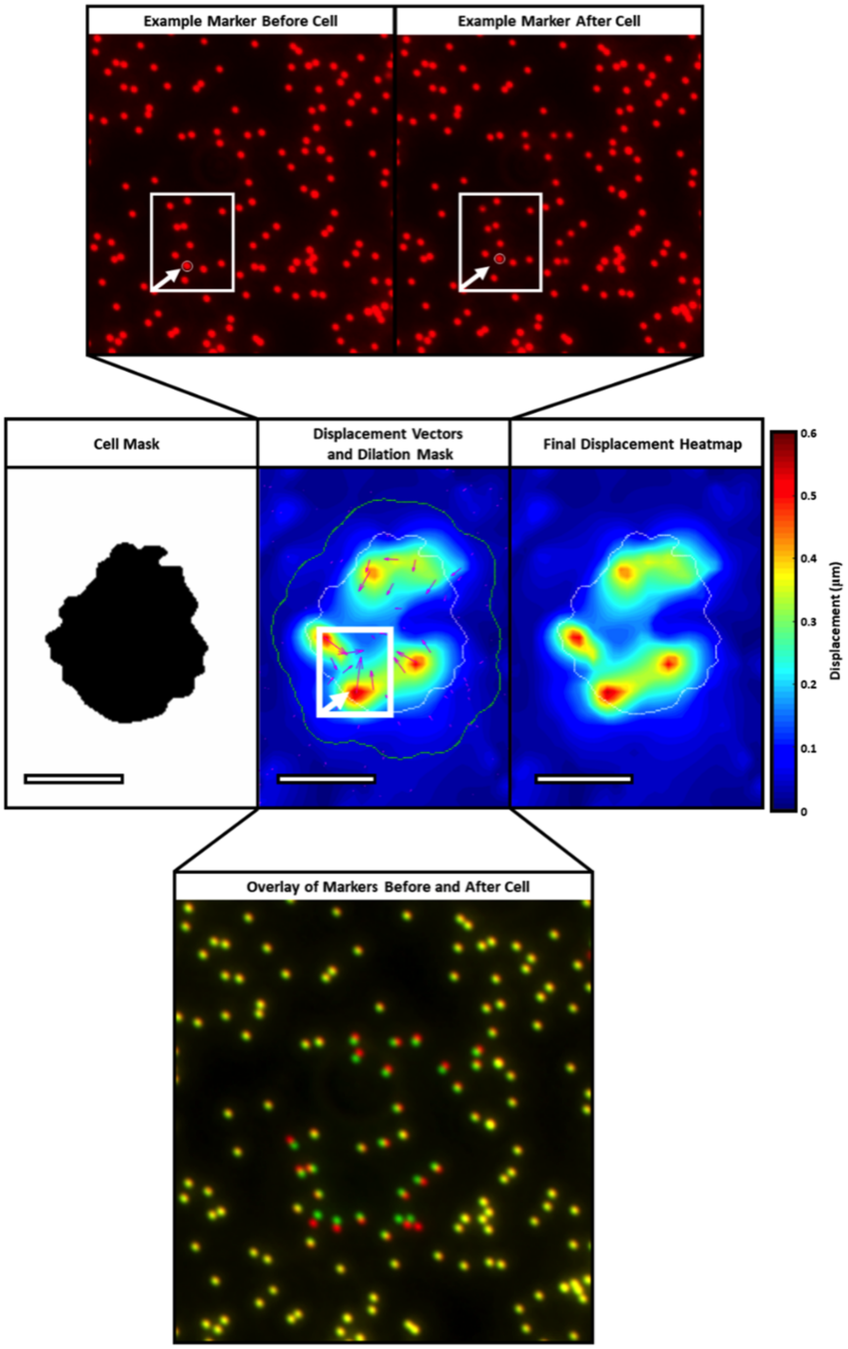

Figure 3. Representative images of cell-induced fluorescent marker displacement. Representative fluorescent images of a 10 kPa gel with a single layer of 0.5 μm fluorescent beads before and after the addition of a neutrophil. **(Top)** Fluorescent markers before (left) and after (right) cell addition with a representative bead (white) shown moving from the fixed point. **(Middle)** The cell mask and displacement heatmap with and without vectors with the same representative bead highlighted (white) and the dilation field outlined in green. Scale bars are 10 μm. **(Bottom)** Overlay of the bead conformation before cell addition (red) and after cell addition (green), highlighting cell-induced marker displacement.
